# ARF1 recruits RAC1 to leading edge in neutrophil chemotaxis

**DOI:** 10.1186/s12964-017-0193-y

**Published:** 2017-10-02

**Authors:** Yuichi Mazaki, Yasuhito Onodera, Tsunehito Higashi, Takahiro Horinouchi, Tsukasa Oikawa, Hisataka Sabe

**Affiliations:** 1 Department of Cellular Pharmacology, Graduate School of Medicine, Hokkaido University, Sapporo, Japan; 2 Department of Molecular Biology, Graduate School of Medicine, Hokkaido University, Sapporo, Japan

**Keywords:** Chemotaxis, ARF1, GBF1, RAC1

## Abstract

**Background:**

The small GTPase ARF1 mediates membrane trafficking mostly from the Golgi, and is essential for the G protein-coupled receptor (GPCR)-mediated chemotaxis of neutrophils. In this process, ARF1 is activated by the guanine nucleotide exchanger GBF1, and is inactivated by the GTPase-activating protein GIT2. Neutrophils generate the Gβγ-PAK1-αPIX-GIT2 linear complex during GPCR-induced chemotaxis, in which αPIX activates RAC1/CDC42, which then employs PAK1. However, it has remained unclear as to why GIT2 is included in this complex.

**Results:**

We investigated the association between ARF1 and RAC1/CDC42 during the *f*MLP-stimulated chemotaxis of HL60 cells. We found that the silencing of *GBF1* significantly impaired the recruitment of RAC1 to the leading edges, but not PAK1, αPIX, RAC2, or CDC42. A significant population of RAC1 colocalized with ARF1 at the leading edges in stimulated cells, whereas *f*MLP activated both ARF1 and ARF5. Consistently, the silencing of *ARF1*, but not *ARF5*, impaired the recruitment of RAC1, whereas the silencing of *RAC1* did not affect the recruitment of ARF1 to the leading edges.

**Conclusions:**

Our results indicated that the activation of ARF1 triggers the plasma membrane recruitment of RAC1 in GPCR-mediated chemotaxis, which is essential for cortical actin remodeling. Thus, membrane remodeling at the leading edges appears to precede actin remodeling in chemotaxis. Together with the fact that GIT2, which inactivates ARF1, is an integral component of the machinery activating RAC1, we proposed a model in which the ARF1-RAC1 linkage enables the regulation of ARF1 by repetitive on/off cycles during GPCR-mediated neutrophil chemotaxis.

## Introduction

Neutrophils are rapidly polarized upon the detection of a chemoattractant gradient, and start to migrate toward the chemoattractant source. Such directional cell migration requires a complex but well organized series of intracellular events, such as cytoskeleton remodeling, and membrane trafficking and remodeling. Most chemoattractants, including *N*-formyl-Met-Leu-Phe (*f*MLP), bind to their cognate G protein-coupled receptors (GPCRs), and this binding then releases the Gα subunit and the Gβγ heterodimer from heterotrimeric G proteins to transmit the downstream signals [1, 2]. αPIX is a Dbl-family guanine nucleotide exchange factor (GEF) for RAC1 and CDC42 [3], whereas p21-activating protein 1 (PAK1) is a downstream effector of activated RAC1 and CDC42 [4]. In neutrophil chemotaxis, Gβγ binds to PAK1, which then binds to αPIX, thus forming the linear complex of Gβγ-PAK1-αPIX, which regulates the activities of the RHO-family GTPases, to remodel the actin-based cytoskeletal structure upon GPCR signaling [5].

During GPCR-induced neutrophil chemotaxis, RAC1 primarily controls directional sensing, in which RAC1-deficient neutrophils frequently generate multi-head leading edges during chemotaxis [6]. Likewise, CDC42-deficient neutrophils also generates similar multi-head leading edges [7]. On the other hand, RAC2 appears to be crucial for actin polymerization at the leading edges, as RAC2-deficient neutrophils showed significant defects in actin polymerization upon GPCR stimulation, and thereby a loss of chemokinesis [6, 8].

Membrane remodeling is another essential part of neutrophil chemotaxis. ARF-family GTPases are primarily engaged in membrane trafficking and remodeling, and are hence crucial to higher order cellular functions, including cell motility [9–11]. ARF1 is primarily involved in membrane and vesicle trafficking from the Golgi [12, 13]. We previously showed that GIT2, which is a GTPase-activating protein (GAP) for ARF1, binds to αPIX to form a linear complex of Gβγ-PAK1-αPIX-GIT2 [14]. This complex, as well as GIT2 on its own, was crucial for the suppressive control of ARF1 activity during GPCR signaling. Interestingly, GIT2 was moreover found to be crucially involved in the efficient recruitment and activation of RAC1 upon GPCR stimulation, whereas CDC42 and RAC2 were almost unaffected by the GIT2 deficiency [14]. As a result, GIT2-deficient neutrophils lose their directional persistency in GPCR-mediated chemotaxis, whereas the rates of actin-cytoskeletal polymerization and cell migration are almost unaffected. Furthermore, the suppressive control of ARF1 by GIT2 is important for the proper production of superoxide, both in time and in direction, during GPCR-mediated neutrophil chemotaxis [14].

Processes activating ARF1 appear to be important for several aspects of neutrophil chemotaxis. We identified that among the different ARFGEFs, GBF1 is most crucial for the activation of ARF1 in neutrophils upon GPCR stimulation, in which GBF1 first translocates from the Golgi to the leading edges, and then recruits ARF1 and GIT2 to the leading edges [15]. In this process, the expression of a dominant-active form of ARF1, namely ARF1(Q71L), was sufficient to recruit GIT2. *GBF1* silencing impaired the directional migration, whereas cell migration rates were not notably affected; and moreover, this silencing, as well as the expression of the dominant-negative form of ARF1, ARF1(T31 N), frequently generated multi-head leading edges during chemotaxis, similar to those observed previously upon the deficiency of RAC1 or CDC42 [15]. Thus, a close association appears to exist between ARF1 and these RHO-family GTPases in GPCR-mediated neutrophil chemotaxis, with regard to their plasma membrane recruitment and activation. Furthermore, *GBF1* silencing was found to affect the proper production of superoxide upon GPCR stimulation, which might be a reflection of the fact that GBF1 is required to recruit GIT2 to the leading edges [15].

ARF-family GTPases may function through their cycles of activation and inactivation. For example, expression of either the GTP hydrolysis-deficient mutant or the GDP-bound mutant of ARF1 both blocked the functions of ARF1 associated with ER-Golgi transport [16]. However, the molecular mechanisms by which the activation processes of the ARF-GTPases are coupled with the inactivation processes remain unclear. We show here that ARF1 activation recruits RAC1 to the leading edges of GPCR-stimulated neutrophils, and propose that this link generates a system in which ARF1 activation is automatically coupled with its inactivation process at the leading edges during GPCR-stimulated chemotaxis of neutrophils.

## Results and Discussion

### Recruitment of Gβγ, αPIX, and PAK1 to leading edges occur independent of the GBF1

**Fig. 1:**
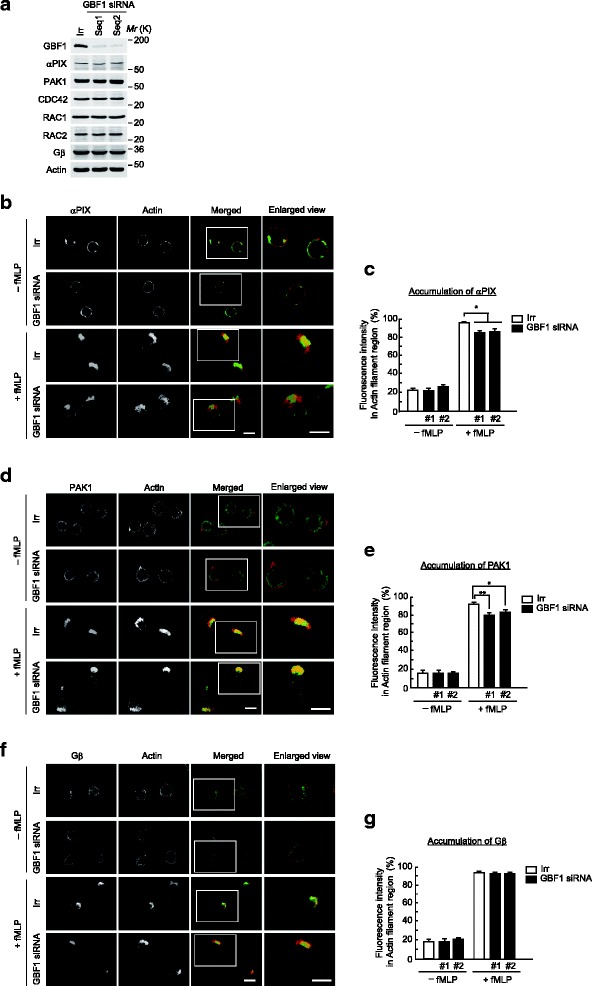
Independence of GBF1 in the translocation of PAK1, αPIX, and Gβγ to the leading edges. (**a**) Expression pattern of proteins in cells treated with *GBF1* siRNAs . Cells transfected with siRNA against *GBF1* or an irrelevant RNA duplex (Irr) were analyzed for expression of the indicated proteins by immunoblotting of the lysates (10 μg each). Data are representative of three independent experiments. (**b**-**g**) Subcellular localization of αPIX, PAK1, and Gβ. Differentiated HL-60 cells, transfected with *GBF1* siRNA or Irr, were incubated with or without *f*MLP for 15 min, and subjected to anti-αPIX (**b**), anti-PAK1 (**d**), or anti-Gβ immunostaining (**f**), and percentages of αPIX molecules (**c**), PAK1 molecules (**e**), or Gβ molecules (**g**) translocated to the leading edges in *f*MLP-stimulated cells were calculated. F-actin was visualized by Texas Red-phalloidin. Data are representative images of three independent experiments (**b**, **d**, and **f**), and >25 cells were analyzed in three independent experiments (**c**, **e**, and **g**). Error bars, SEM (**c**, **e**, and **g**). ** *p* < 0.01 and * *p* < 0.05 compared with the Irr control. Bars, 10 μm (**b**, **d**, and **f**)

Silencing of *GBF1* in HL-60 cells frequently generated multi-head leading edges during *f*MLP-induced chemotaxis, similar to those observed upon the inhibition of RAC1 or CDC42 [15]. We have shown that *GBF1* small interfering RNA (siRNA) treatment causes loss of the polarized accumulation of GIT2 at the leading edges of *f*MLP-stimulated HL-60 cells, in which a greater than 50% decrease in the accumulation of GIT2 at actin-rich leading edges was observed compared with cells treated with a control irrelevant siRNA [15]. GIT2 forms a complex with αPIX and PAK1, which are an activator and an effector of RAC1, respectively. αPIX and PAK1 accumulate at actin-rich leading edges upon *f*MLP stimulation, whereas these proteins mostly localize around the cell periphery in unstimulated neutrophils [14] (also see Fig. 1b and d). We hypothesized that the lack of GIT2 recruitment upon *GBF1* silencing may impair the recruitment of αPIX and PAK1, thus causing the dysfunction of RAC1 at the leading edges. We then suppressed the expression of GBF1 protein by siRNA method in differentiated HL-60 cells. We found that two siRNA sequences of *GBF1* block expression of GBF1 protein without notable suppression of others protein expression in differentiated HL-60 cells (Fig. 1a). However, unlike in the case of GIT2, *GBF1* silencing decreased the accumulation of αPIX and PAK1 at the leading edges only by approximately 10% in *f*MLP-stimulated HL-60 cells (Fig. 1b-e).

Gβγ forms a complex with GIT2, via αPIX and PAK1; GIT2 deficiency caused a substantial loss of the polarized accumulation of Gβγ at the leading edges of GPCR-stimulated neutrophils (> 50% decrease, Mazaki et al., 2006). On the other hand, a substantial fraction of Gβγ appeared to localize to the cytosol, rather than to the cell periphery, in the unstimulated neutrophils [14] (also see Fig. 1f). No significant reduction was observed by *GBF1* silencing in the recruitment of Gβγ leading edges upon *f*MLP stimulation (Fig. 1f and g). Collectively, it is likely that although GBF1 is crucial for the recruitment of GIT2 to leading edges upon GPCR signaling, the recruitment of Gβγ, αPIX, and PAK1 to leading edges is substantially independent of the GBF1-GIT2 axis, despite the fact that these three proteins form a complex with GIT2 in GPCR signaling.

### GBF1 is required for recruitment and activation of RAC1

**Fig. 2:**
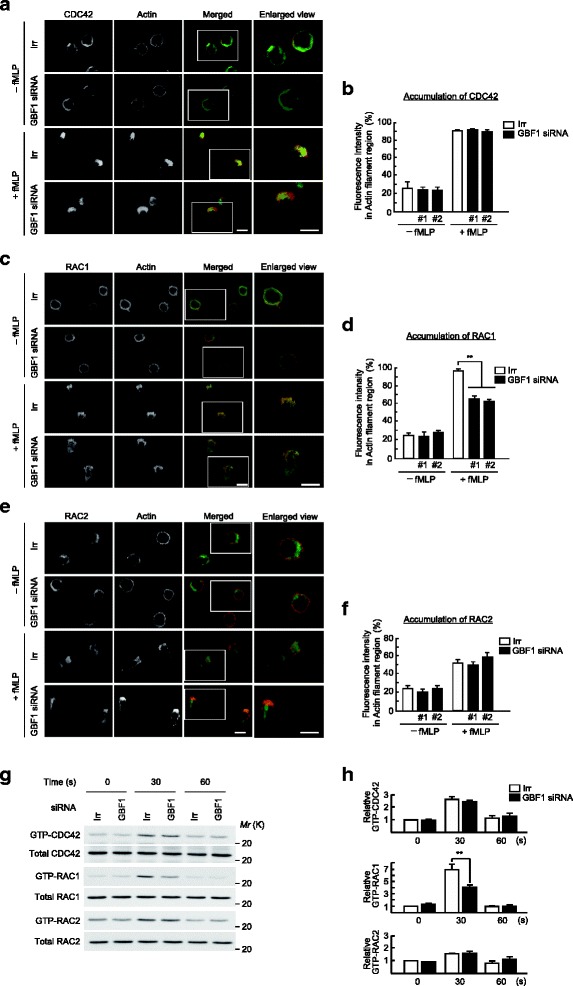
Requirement of GBF1 in RAC1 activity and its translocation to the leading edges. (**a**-**f**) Subcellular localization of CDC42, RAC1, or RAC2. Differentiated HL-60 cells, transfected with *GBF1* siRNA or an irrelevant RNA duplex (Irr), were incubated with or without *f*MLP for 15 min, and subjected to anti-CDC42 (**a**), anti-RAC1 (**c**), or anti-RAC2 immunostaining (**e**), and percentages of CDC42 molecules (**b**), RAC1 molecules (**d**), and RAC2 molecules (**f**) translocated to the leading edge in *f*MLP-stimulated cells were calculated. F-actin was visualized by Texas Red-phalloidin. Data are representative images of three independent experiments (**a**, **c**, and **e**), and >25 cells were analyzed in three independent experiments (**b**, **d**, and **f**). (**g** and **h**) Activities of CDC42, RAC1, and RAC2. Activities of CDC42, RAC1, and RAC2 were measured by GST-PBD pulldown coupled with the indicated antibodies. Each lower panel represents immunoblots of total cell lysates (5 μg) by the indicated antibodies. Data are representative of three independent experiments (**g**), and were analyzed in three independent experiments (**h**). In h, values for Irr control at 0 s are considered 1. Error bars, SEM (**b**, **d**, **f** and **h**). ** represents a statistical difference from the Irr control (*p* < 0.01). Bars, 10 μm (**a**, **c**, and **e**)

RAC1 and CDC42 are also recruited to the leading edges upon GPCR stimulation of neutrophils [17], whereas these proteins mostly localized around the cell periphery in unstimulated cells (Fig. 2a and c). We next examined the effects of *GBF1* silencing on these proteins, and found that the silencing of *GBF1* significantly impaired the recruitment of RAC1 to the leading edges (decreased by 30%–40%), but not CDC42 (Fig. 2a-d). *GBF1* silencing caused no significant reduction in RAC2 accumulation at the leading edges (Fig. 2e and f). Moreover, *GBF1* silencing significantly suppressed the *f*MLP-induced activation of RAC1 in HL-60 cells, as measured 30 s after the stimulation, whereas this silencing did not notably affect the activation of CDC42 and RAC2 (Fig. 2g and h). These results indicated that GBF1 is linked to RAC1, rather than RAC2 or CDC42, in the GPCR signaling of neutrophils.

### ARF1 is required for recruitment and activation of RAC1

**Fig. 3:**
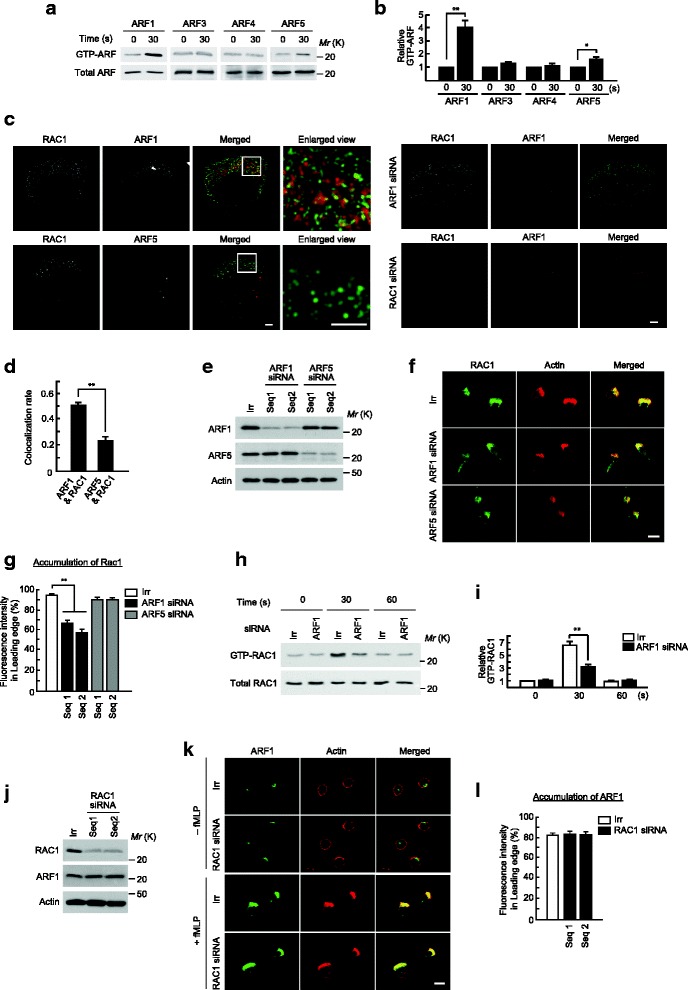
Requirement of ARF1 in RAC1 activity and its translocation to the leading edges. (**a** and **b**) Activity of ARFs in differentiated HL-60 cells after *f*MLP stimulation. Activities of class I and II ARFs were measured by GST-GGA3 pulldown coupled with the indicated antibodies. Each lower panel represents immunoblots of the total cell lysates (5 μg) by the indicated antibodies. Data are representative of three independent experiments (**a**), and were analyzed in three independent experiments (**b**). In b, values of each GTP-ARF at 0 s are considered 1. ** *p* < 0.01 and * *p* < 0.05 compared with each GTP-ARF at 0 s. (**c** and **d**) Subcellular localization of RAC1, ARF1, and ARF5 after *f*MLP stimulation. Differentiated HL-60 cells were incubated with *f*MLP for 5 min, and subjected to immunostaining analysis, using high-resolution SIM. Specificities of the anti-ARF1 antibody and the anti-RAC1 antibody were confirmed by *ARF1* or *RAC1* siRNA-treatment of cells (**c**) Bars, 2 μm. Pearson’s correlation coefficients of the intracellular colocalization of these proteins, as indicated, were estimated from >10 cells (**d**). (**e**) Suppression of ARF1 or ARF5 by siRNAs in differentiated HL-60 cells. Cells transfected with siRNA against *ARF1*, *ARF5*, or an irrelevant RNA duplex (Irr) were analyzed for the expression of the indicated proteins by immunoblotting of the lysates (10 μg each). Data are representative of three independent experiments. (**f** and **g**) Subcellular localization of RAC1. Differentiated HL-60 cells, transfected with siRNA against *ARF1*, *ARF5*, or Irr, were incubated with *f*MLP for 15 min, and subjected to anti-RAC1 immunostaining (**f**), and percentages of RAC1 molecules translocated to the leading edges in *f*MLP-stimulated cells were calculated (**g**). (**h** and **i**) Activities of RAC1. Activities of RAC1 were measured by GST-PBD pulldown coupled with the anti-RAC1 antibodies. Each lower panel represents immunoblots of total cell lysates (5 μg) by the anti-RAC1 antibodies. Data are representative of three independent experiments (**h**), and were analyzed in three independent experiments (**i**). In i, values for Irr control at 0 s are considered 1. (**j**) Suppression of RAC1 by siRNAs in differentiated HL-60 cells. Cells transfected with siRNA against *RAC1* or Irr were analyzed for expression of the indicated proteins, by immunoblotting of the lysates (10 μg each). Data are representative of three independent experiments. (**k** and **l**) Subcellular localization of ARF1. Differentiated HL-60 cells, transfected with *RAC1* siRNA or Irr, were incubated with or without *f*MLP, as indicated, and subjected to anti-ARF1 immunostaining (**k**), and percentages of ARF1 molecules translocated to the leading edges in *f*MLP-stimulated cells were calculated (**l**). F-actin was visualized by Texas Red-phalloidin. Data are representative images of three independent experiments (**f**, and **k**), and >25 cells were analyzed in three independent experiments (**g** and **l**). Error bars, SEM (**b**, **d**, **g, i** and **l**). ** represents a statistical difference from Irr (*p* < 0.01) (**g** and **i**). Bars, 10 μm (**f** and **k**)

We then addressed the mechanism by which GBF1 is involved in the recruitment of RAC1 to the leading edges. ARF1 may not be the sole target of GBF1 [18, 19]. In fact, in addition to ARF1, *f*MLP stimulation activated ARF5 in HL-60 cells (these cells express ARF1, 3, 4, and 5) (Fig. 3a and b). We then investigated the possible colocalization of ARF1 and ARF5 with RAC1 upon *f*MLP stimulation. ARF1 and RAC1 each showed a punctate distribution at the leading edges of stimulated HL-60 cells, in which a significant fraction of ARF1 and RAC1 are colocalized (Fig. 3c and d). ARF5 also showed a punctate distribution at the leading edges, but its colocalization with RAC1 appeared to be very limited compared with that of ARF1 (Fig. 3c and d). We then found that silencing of *ARF1* significantly inhibited the recruitment of RAC1 to the leading edges upon *f*MLP stimulation of HL-60 cells (~40% inhibition), whereas the silencing of *ARF5* did not affect RAC1 recruitment at all (Fig. 3f and g). Thus, ARF1, but not ARF5, appeared to be crucial for the recruitment of RAC1, whereas ARF1 and ARF5 are both activated under this condition. Moreover, *ARF1* silencing significantly suppressed the *f*MLP-induced activation of RAC1 in HL-60 cells, as measured 30 s after the stimulation (Fig. 3h and i). We also analyzed whether the silencing of *RAC1* affects the recruitment of ARF1 to the leading edges upon *f*MLP stimulation, and found that RAC1 was not at all required for the recruitment of ARF1 (Fig. 3k and l). Taken together, it is conceivable that the activation of ARF1 by GBF1 plays an important role in the recruitment of RAC1 to the leading edges and the activation of RAC1 upon the GPCR signaling of neutrophils.

**Fig. 4:**
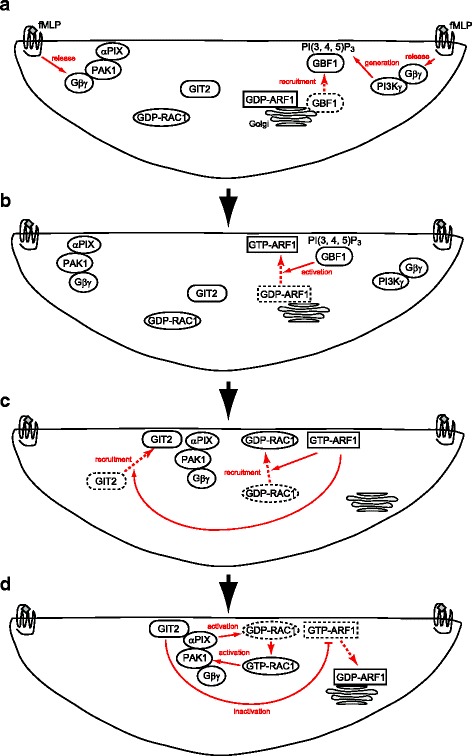
A model for the cyclic activation and inactivation of ARF1 during GPCR-stimulated neutrophil chemotaxis. (**a**) RAC2 is crucial for the generation of actin-based leading edges in neutrophils, whereas RAC1 is important for directional migration. Polarized activation of GPCR at cell surface areas facing a chemoattractant gradient, such as by *f*MLP, releases the Gβγ subunit, which leads to RAC2 activation via the production of PI(3, 4, 5)P_3_ upon activation of PI3Kγ by Gβγ, to generate the actin-based leading edge [2, 31]. GBF1 is recruited to the leading edge from the Golgi, also by the Gβγ-PI3Kγ-mediated production of PI(3, 4, 5)P_3_. Gβγ proteins may furthermore form a complex with PAK1-αPIX at the leading edges. (**b**) GBF1 then recruits and activates ARF1 at the leading edges, although the mechanism by which ARF1 is recruited by GBF1 remains unknown. (**c**) The activated ARF1 then recruits RAC1 and GIT2 to the leading edges. (**d**) RAC1 is activated by αPIX and functions with PAK1. Integration of GIT2 into the Gβγ-PAK1-αPIX complex provides a mechanism by which ARF1 can be inactivated when RAC1 becomes activated and functional. This system may enable the cyclic activation and inactivation of ARF1 for the repetitive recruitment of RAC1 molecules into the growing leading edges, culminating in the directional migration of GPCR-stimulated neutrophils. On the other hand, GTP-ARF1 and GTP-RAC1 need to function together to perform certain cellular functions (see *Text*). Thus, ARF1 might not always be inactivated when RAC1 is activated, and the timing of ARF1 inactivation by the Gβγ-PAK1-αPIX-GIT2 complex might be controlled by unknown mechanisms

The fact that GIT2, which is a GAP for ARF1, is an integral component of the Gβγ-PAK1-αPIX complex [14] has been enigmatic, as this complex apparently forms primarily to activate and assist in the functioning of RAC1 and/or CDC42. In the present study, we found that activation of ARF1 by GBF1 at the leading edges of cells recruits RAC1 to the same leading edges; we hence propose a model in which ARF1 activity is regulated by repetitive on/off cycles during GPCR-mediated neutrophil chemotaxis (Fig 4). Our model explains that the inclusion of GIT2 as a member of the Gβγ-PAK1-αPIX complex provides a system by which ARF1 activity can be repetitively regulated between its activation and inactivation cycles, concurrently with plasma membrane protrusion and the formation of leading edges. In other words, ARF1 on its own appears to generate this system by recruiting RAC1, in order to perform directional cell migration.

Our results demonstrated that ARF1 activation precedes RAC1 activation and function in cell migration. This notion is consistent with the concept previously proposed by Donaldson, in which ARF-mediated membrane remodeling is a prerequisite for actin-cytoskeletal remodeling, which is mediated by RHO-family GTPases [20].

ARF1 might not always be inactivated when RAC1 is activated. Wiskott-Aldrich syndrome protein (WASP)-family verprolin homologous protein (WAVE) regulatory complex (WRC) is crucial for the dynamic regulation of the structure of leading edges, by promoting membrane ruffling and lamellipodia formation [21]. Activated RAC1 is essential for WRC function [21]. It was reported that the binding affinity of GTP-RAC1 to WRC is relatively low, and that although GTP-ARF1 also binds weakly to WRC, GTP-ARF1 assists the binding of RAC1 to WRC [22]. Consistently, cooperation of ARF1 and RAC1 was shown to be necessary to induce WAVE-induced actin polymerization [23]. Thus, our results may have also illustrated a process in which these two small GTPases are both activated and function cooperatively with each other.

Then, an important question remains as to how the timing of the inactivation of ARF1 by the Gβγ-PAK1-αPIX-GIT2 complex is regulated. αPIX can bind directly to the plasma membrane via its pleckstrin homology domain, and this binding recruits PAK1 to the plasma membrane [5, 24]. As αPIX and PAK1, as well as GIT2, are accumulated at leading edges upon GPCR stimulation, it is likely that the Gβγ-PAK1-αPIX-GIT2 complex is formed at the leading edges. On the other hand, activation of ARF1 by GBF1 occurs independently of this complex upon GPCR stimulation, as we have shown that GBF1 is activated by a product of phosphatidylinositol-3-phosphate kinase γ (PI3Kγ) [15], which is activated via its binding to Gβγ [2]. Thus, a more precise picture of the inactivation process of ARF1 by the Gβγ-PAK1-αPIX-GIT2 complex will be required to understand the nature of neutrophil chemotaxis. Mechanisms by which ARF1 recruits RAC1 also await to be clarified.

## Materials and methods

### Cells

HL-60 cells were obtained from ATCC. HL-60 cells were cultured in RPMI 1640 medium supplemented with 10% fetal bovine serum (Gibco) and 2 mM L-glutamine. For differentiation into neutrophil-like cells, cells were cultured in the presence of 1.25% dimethyl sulfoxide for 6 days, as described previously [25].

### Antibodies and chemicals

Antibodies were purchased from the following commercial sources: mouse monoclonal antibody against ARF1 and ARF5 (Abcam), RAC1 (Millipore), CDC42 (Santa Cruz Biotechnology), αPIX (Abnova), actin (Sigma); rabbit polyclonal antibodies against ARF3, ARF4, and RAC1 (Abcam), RAC2 and Gβ (Millipore), and PAK1 (Santa Cruz Biotechnology). Donkey antibody against mouse and rabbit IgG, conjugated with horseradish peroxidase, were from Jackson ImmunoResearch Laboratories. Goat antibodies against mouse and rabbit IgGs, conjugated with Alexa Fluor 488 or Alexa Fluor 555, and phalloidins, conjugated with Texas Red, were from Invitrogen. All other chemical reagents were purchased from Sigma and Nacalai, unless otherwise stated.

### Transfections

Transfections were performed as described previously [15]. For the transfection of siRNA, 3 μg of siRNAs each specific to GBF1, ARF1, ARF5, and RAC1, or an irrelevant RNA duplex (siCONTROL, RISC-free siRNA1; Dharmacon) were used. GBF1 siRNA targeting sequences were as described previously [15]. ARF1 and ARF5 siRNA targeting sequences were 5′-TGACAGAGAGCGTGTGAAC-3′ (ARF1 sequence 1), 5′-ACCGUGGAGUACAAGAACA-3′ (ARF1 sequence 2), 5′- TCTGCTGATGAACTCCAGA-3′ (ARF5 sequence 1) and 5′- CCATAGGCTTCAATGTAGA-3′ (ARF5 sequence 2), as described previously [26]. RAC1 siRNA targeting sequences were 5′- AGACGGAGCTGTAGGTAAA-3′ (RAC1 sequence 1) and 5′-TAAGGAGATTGGTGCTGTA-3′ (RAC1 sequence 2), as described previously [27].

### Immunofluorescence microscopy

Immunofluorescence microscopy was performed as described previously [15]. Briefly, differentiated HL-60 cells were attached to coverslips in Hank’s balanced salt solution (HBSS) containing 20 mM 4-(2-hydroxyethyl)-1-piperazineethanesulfonic acid (pH 7.2) and 0.1% bovine serum albumin. Coverslips were then placed on Dunn chambers (Hawksley), and incubated for the indicated times at 37 °C. Acquisition of confocal images using a laser-scanning microscope (FV500; Olympus) was performed as previously described [14]. Each experiment was performed three times, in each of which more than 50 cells were analyzed, and representative images are shown in each figure. High-resolution structured illumination (SIM) microscopy analysis was performed to analyze the intracellular colocalization of RAC1 with ARF1 or with ARF5 using an N-SIM microscope (Nikon) and NIS-elements software (Nikon), as described previously [28].

### Small GTPase activities

For measurement of small GTPase activities, 1 × 10^6^ cells were washed, and preincubated in HBSS for 5 min at 37 °C, and then stimulated with 100 nM *f*MLP or left untreated for the indicated times in the same solution at 37 °C. Cells were then solubilized, and GTP-bound class I and II ARFs were pulled-down using 50 μg of glutathione S-transferase (GST)-GGA3_1–226_ [29]. GTP-bound CDC42, RAC1, and RAC2 were pulled-down using GST-PBD [30]. Amounts of these GTPases in total cell lysates were simultaneously determined by immunoblotting using their antibodies. Small GTPase activities were measured by a densitometer (GT-X770 scanner; Epson) using Image version 1.50i software (National Institutes of Health, Bethesda, MD).

### Statistical analysis

For all experiments, differences between groups were calculated by Tukey-Kramer test.

## Abbreviations

*f*MLP: *N*-formyl-Met-Leu-Phe peptide
GAP: GTPase-activating protein
GEF: Guanine nucleotide exchanging factor
GPCR: G protein-coupled receptor
GST: Glutathione *S*-transferase
PAK1: p21-activating protein kinase 1
SIM: Structured illumination microscopy
siRNA: small interfering RNA
WRC: WAVE regulatory complex
